# Therapeutic Advancements in Psoriasis and Psoriatic Arthritis

**DOI:** 10.3390/jcm14041312

**Published:** 2025-02-16

**Authors:** Robin C. Yi, Maya Akbik, Logan R. Smith, Yael Klionsky, Steven R. Feldman

**Affiliations:** 1 Center for Dermatology Research, Department of Dermatology, Wake Forest University School of Medicine, Winston-Salem, NC 27157, USA; lrsmith1@usf.edu (L.R.S.); sfeldman@wakehealth.edu (S.R.F.); 2 Medical College of Georgia at Augusta University, Augusta, GA 30912, USA; makbik@augusta.edu; 3 Department of Internal Medicine, Division of Rheumatology, Wake Forest University School of Medicine, Winston-Salem, NC 27101, USA; yklionsk@wakehealth.edu; 4 Department of Pathology, Wake Forest University School of Medicine, Winston-Salem, NC 27157, USA; 5 Department of Social Sciences & Health Policy, Wake Forest University School of Medicine, Winston-Salem, NC 27157, USA

**Keywords:** psoriasis, psoriatic arthritis, biologics, oral small molecules, new therapies

## Abstract

**Background:** Within the past few years, many new therapies have emerged for psoriasis and psoriatic arthritis (PsA). Current topical therapies—including corticosteroids, vitamin D analogs, tapinarof, and roflumilast—remain the mainstay for mild disease, while oral systemic and biologic options are for moderate to severe cases. Biologics—such as Tumor necrosis factor-alpha (TNF-alpha), Interleukin 12/23 (IL-12/23), Interleukin-17 (IL-17), and Interleukin-23 (IL-23)—have revolutionized care by providing highly effective and safer alternatives. Oral small molecules, including Janus kinase (JAK) and tyrosine kinase 2 (TYK2) inhibitors, further expand the therapeutic options. **Objectives**: The goal for this review article was to examine current and latest treatments for psoriasis and PsA and discuss whether these emerging therapeutic options address the unmet needs of current treatments. **Methods**: The search for this review article included PubMed, Google Scholar, and ClinicalTrials.gov for relevant articles and current clinical trials using keywords. **Results:** A wide range of novel psoriatic and PsA therapies are currently undergoing clinical trials. These include selective JAK inhibitors, TYK2 inhibitors, retinoic acid-related orphan receptor (RORγT) inhibitors, oral IL-23 receptor inhibitors, oral IL-17A inhibitors, nanobody products, sphingosine-1-phosphate (S1P1R) antagonists, A3 adenosine receptor (A3AR) agonists, heat shock protein (HSP) 90 inhibitors, and rho-associated protein kinases (ROCK-2) inhibitors. **Conclusions:** These different mechanisms of action not only expand treatment options but may offer potential solutions for patients who do not achieve adequate response with existing therapies. However, the safety and contraindications of these newer agents remain an important consideration to ensure appropriate patient selection and minimize potential risks. Certain mechanisms may pose increased risks for infection, cardiovascular manifestations, malignancy, or other immune-related adverse events, necessitating careful monitoring and individualized treatment decisions. Ongoing clinical research aims to address unmet needs for patients who do not respond to previous agents to achieve sustained remission, monitor long-term safety outcomes, and assess patient preferences for delivery, including a preference for oral delivery. Oral IL-23 inhibitors hold potential due to their robust safety profiles. In contrast, oral IL-17 inhibitors and TYK-2 inhibitors are effective but may present side effects that could impact their acceptability. It is essential to balance efficacy, safety, and patient preferences to guide the selection of appropriate therapies.

## 1. Introduction

Psoriasis and psoriatic arthritis (PsA) are interconnected diseases within the broader domain of psoriatic disease [1]. Both conditions share common immunopathological mechanisms involving pro-inflammatory cytokines, which drive abnormal immune responses to skin, joint, bone, and vascular inflammation [1]. For mild psoriasis, topical corticosteroid therapies have been the gold standard of treatment, while ultraviolet (UV) phototherapy has helped with extensive psoriatic manifestations [2]. Historically, oral systemic treatment, such as methotrexate (MTX) and cyclosporine (CyA), helped to broaden disease control for patients not adequately responsive to topicals and phototherapy. Some side effects of these oral anti-metabolites warrant careful laboratory monitoring due to hepatotoxicity and nephrotoxicity, respectively. Within the past few years, advancements in the understanding of the pathophysiology of psoriasis and PsA have guided the evolution of treatment options. The introduction of biologics marked a turning point in therapeutic guidelines for both conditions, as these agents specifically target key cytokines involved by targeting specific inflammatory pathways, leading to positive responses with fever systemic side effects. Biologics—including Tumor necrosis factor (TNF) inhibitors, interleukin-17 (IL-17) inhibitors, and interleukin-23 (IL-23) inhibitors—have revolutionized the treatment landscape by providing more targeted and effective options. Contraindications of biologics include increased cardiovascular events with IL-23 inhibitors and onset or worsening of inflammatory bowel disease with IL-17 inhibitors [3,4]. Therefore, emerging biologics and oral small molecules (OSMs) are set to further expand treatment options by providing new targeted approaches to address the underlying pathways of both conditions. This article examines the current and latest treatments for psoriasis and PsA and discusses whether these emerging therapeutic options address the unmet needs of current treatments. We first provide a narrative review of current treatments, providing an understanding of current unmet needs in psoriasis and PsA, and then report a review of treatments in development and how they may or may not meet those needs.

### 1.1. Current Treatments for Psoriasis and Psoriatic Arthritis

Therapeutic advancements in psoriasis and PsA have provided various treatment options tailored to disease severity and individual needs. The progressive understanding of the shared immunopathophysiology of psoriasis and PsA has enabled the development of targeted therapies that address the underlying inflammation. For psoriasis, initial topical treatments include coal tar, which was used for hundreds of years for its anti-inflammatory properties [5]. Phototherapy, particularly with natural sunlight and later refined to UV light therapy, emerged as another cornerstone treatment for systemic disease [2]. When considering phototherapy options for psoriasis, narrow-band ultraviolet B (NB-UVB) phototherapy is often preferred due to its lower risk profile compared to other modalities [6]. Unlike psoralen plus UVA (PUVA) therapy, NB-UVB has a reduced risk of skin cancer, as it does not require a photosensitizing agent and has been associated with a lower cumulative risk of non-melanoma skin cancer with decreased systemic effects. Ultraviolet A (UVA) therapy is an option without psoralen but is less commonly used for psoriasis due to lower efficacy from the absence of the psoralen, a photosensitizing agent. However, avoiding psoralen use reduces the risk of systemic side effects, such as hepatotoxicity and nausea, making it a consideration for patients who cannot tolerate PUVA [7]. While these two therapies are still widely used, the introduction of topical corticosteroids in the 1950s revolutionized psoriasis treatment, establishing them as the gold standard for managing inflammation and symptoms in localized disease [8]. PsA was later recognized as a distinct inflammatory arthritis associated with psoriasis. For PsA therapy, nonsteroidal anti-inflammatory drugs (NSAIDs) are utilized for symptomatic management of mild PsA-related arthralgias, while disease-modifying antirheumatic drugs (DMARDs) are the initial therapy for addressing underlying disease activity and preventing progression.

### 1.2. Topical Therapies

Topical therapies play a central role in the management of mild to moderate psoriasis, particularly in cases of limited disease (Table 1). Topical corticosteroids are first-line psoriasis treatment and are often used alone or in combination with other systemic agents to enhance efficacy while minimizing side effects. Vitamin D analogs, such as calcipotriene, offered an effective steroid-sparing option when introduced in the 1990s, providing a targeted approach to modulate skin cell growth and reduce inflammation [9]. In recent years, other steroid alternative agents such as tapinarof, an aryl hydrocarbon receptor modulator, and roflumilast, a phosphodiesterase-4 (PDE4) inhibitor, have expanded topical treatment options since their United States Food and Drug Administration (FDA) approval in 2022 [10]. Tacrolimus and pimecrolimus, calcineurin inhibitors, were FDA-approved for atopic dermatitis in 2000, but are not approved for psoriasis [11]. These topicals are often used off-label for managing facial and intertriginous psoriasis due to their efficacy in thinner skin areas. In two 8-week randomized clinical trials, 65% of patients using 0.1% tacrolimus and 71% of patients using 0.1% pimecrolimus had improvement or were clear for facial and intertriginous psoriasis compared to placebo [12].

### 1.3. Oral Small Molecules (OSMs)

Oral small molecules (OSMs) were introduced for psoriasis in the 1970s, with the approval of methotrexate (MTX) for PsA in the 1980s (Table 2) [16]. MTX was the first disease-modifying antirheumatic drug (DMARDs) to address systemic inflammation and prevent disease progression in both conditions [17]. MTX, a dihydrofolate reductase antagonist, has been the gold standard therapy for decades and requires safety laboratory monitoring due to potential adverse effects such as myelosuppression, hepatotoxicity, and gastrointestinal upset [18]. Cyclosporine (CyA), a calcineurin inhibitor, is another DMARD primarily used for severe, refractory psoriasis, which was FDA-approved in 1997 [19]. Long-term use is limited by risks such as nephrotoxicity, hyperkalemia, hypertension, hyperuricemia, hyperglycemia, neurotoxicity, and hirsutism [20]. Acitretin is an oral vitamin A derivative that was FDA-approved for the treatment of moderate to severe psoriasis in 1996 [21]. Acitretin is often used as a second- or third-line treatment for severe psoriasis refractory to topicals or when the use of immunosuppressants is contraindicated. The adverse effects are hepatotoxicity, teratogenicity, and hyperlipidemia [22]. Janus kinase (JAK) inhibitors, such as tofacitinib (FDA-approved for PsA in 2017) and upadacitinib (FDA-approved for PsA in 2021), have introduced approaches to modulate immune signaling pathways that do not have the renal and hepatic toxicity of CyA and MTX [23]. These newer agents provide effective options for patients with moderate to severe PsA disease who may not respond adequately to conventional DMARDs. Deucravacitinib, a tyrosine kinase 2 (TYK2) inhibitor, was FDA-approved in 2022 for moderate to severe plaque psoriasis [24]. In a randomized clinical trial, 65.1% of patients using deucravacitinib 6 mg daily achieved Psoriasis Area and Severity Index (PASI)-75 at 16-weeks compared to oral apremilast 30 mg twice daily [25]. For PsA, in two randomized phase 2 and 3 clinical trials more patients achieved American College of Rheumatology 20 (ACR-20) at week 16 than with placebo [26]. Adverse events of nasopharyngitis, herpes zoster, tuberculosis, malignancy, hypertension, and hypercholesteremia were observed in these clinical trials [25,26]. Vaccination against herpes zoster is recommended before initiating oral immunosuppressants and JAK inhibitors due to increased risk of herpes zoster reactivation, also in its atypical variants, such as zoster sine herpete [27]. The incidence of malignancy may be higher in elderly patients, particularly those with rheumatoid arthritis (RA) or myelodysplastic syndrome, as well as in individuals receiving prolonged therapy with tofacitinib or ruxolitinib (oral JAK inhibitors) or anti-TNF agents. Studies have suggested that chronic immunosuppression associated with these therapies may contribute to an increased risk of lymphoproliferative disorders, lung cancer, and non-melanoma skin cancers (NMSCs) [28].

### 1.4. Biologic Therapy

Biologic therapies have revolutionized the management of moderate to severe psoriasis and PsA, offering highly effective treatment options with improved safety profiles (Table 3). These systemic therapies work by modulating specific immune pathways involved in the pathogenesis of both conditions. Apart from the withdrawn drugs of alefacept and efalizumab, the introduction of TNF-alpha inhibitors marked the beginning of highly effective biologic therapy [41]. Etanercept was approved for PsA in 2002 and psoriasis in 2004, followed by infliximab, approved for PsA in 2005 and psoriasis in 2006, and adalimumab, approved for PsA in 2005 and psoriasis in 2008 [42,43,44].

Advancements continued with the development of interleukin 12/23 (IL-12/23) inhibitors, such as ustekinumab, approved in 2009 for both psoriasis and PsA [45]. Recent therapies target IL-17, which include secukinumab and ixekizumab. They were both approved for psoriasis and PsA in 2015 and 2016, respectively, while brodalumab, targeting the IL-17 receptor A (IL-17RA), was approved for psoriasis in 2017 [46,47,48]. Bimekizumab, targets IL-17A and F and was approved for both psoriasis and PsA in 2023 [49,50]. Innovation in biologics continued with the development of IL-23 inhibitors. Guselkumab was FDA-approved in 2017, followed by tildrakizumab in 2018, and risankizumab in 2019, all for moderate to severe psoriasis, with additional approvals for PsA in subsequent years [51]. These biologics have transformed treatment, offering durable efficacy and favorable safety profiles. Randomized phase 2 and 3 clinical trials involving IL-23 inhibitors achieved PASI-75 in 60–98% patients at 16 weeks [52]. Safety profiles mostly included mild nasopharyngitis infections, diarrhea, and pruritus [52]. The use of biologics does require lab monitoring and must be used judiciously in immunosuppressed patients. The safety profiles of biologic therapies vary by drug class, with each having specific risks and contraindications. TNF inhibitors (TNFis) commonly cause injection-site reactions, tuberculosis infections, and hepatotoxicity due to their immunosuppressive effects [53]. Patients with a history of chronic or latent infections require careful screening before starting TNFi therapy. Additionally, TNFis are contraindicated in individuals with heart failure or peripheral demyelinating disorders, as these conditions may worsen with treatment [53]. IL-17 inhibitors (IL-17is) are generally well tolerated, but more serious adverse effects include an increased risk of Candida infections, likely due to the role of IL-17 in mucosal immunity [53]. There is also a potential association with inflammatory bowel disease exacerbation and major adverse cardiovascular events, making careful patient selection essential [53]. IL-23 inhibitors (IL-23is) have been linked to an increased risk of cardiovascular events, sepsis, pneumonia, cellulitis, and breast cancer [53]. While these risks remain under investigation, patients with a history of cardiovascular disease or malignancy may require closer monitoring. Understanding the adverse effects and contraindications of each biologic class is critical to optimizing treatment selection and ensuring patient safety.

To mitigate some of the pharmaceutical cost of biologics therapies, pharmaceutical companies are also creating biosimilars, drugs that target either the same cytokines or receptors as the originator biologics. These biosimilars are gradually gaining approval for use in psoriasis and PsA.

**Table 3:** Biologic therapies for treatment of psoriasis and psoriatic arthritis. The biologic drugs discussed are categorized below and placed alongside their FDA approval year.

Biologic Therapies		Year FDA-Approved	Approved for Psoriasis, PsA, or Both	Psoriasis Clinical Trial and PASI75	PsA Clinical Trial and ACR20	Adverse Events
TNF-alpha inhibitor	*Certolizumab pegol*	2008	Both	CIMPASI-1 and -2; week 48: 72.7% [54]	RAPID-PsA; week 24: 58% [55]	Injection-site reactions, tuberculosis infections, and hepatotoxicity. Increased risk of cardiovascular events and lymphoma malignancies. Contraindicated in congestive heart failure and peripheral demyelinating disorders [42,44,46].
	*Etanercept*	2004	Both	LIBERATE; week 16: 39.8% [56]	SEAM-PsA; week 12: 73% [57]	
	*Adalimumab*	2008	Both	REVEAL; week 16: 71% [58]	ADEPT; week 48: 59% [44]	
	*Infliximab*	2006	Both	EXPRESS; week 10: 80% [59]	IMPACT; week 16: 20% [60]	
	*Golimumab*	2009	PsA	GO-VIBRANT; week 14: 59.2% [61]	GO-VIBRANT; week 14: 75.1% [61]	
IL-12/23 inhibitor	*Ustekinumab*	2009	Both	PHOENIX-1; week 12: 81.3% [62]	PSUMMIT-1; week 12: 41% [62]	Injection-site reactions and upper respiratory infections. Increased risk of appendicitis, osteomyelitis, diverticulitis, and gastroenteritis [47].
IL-17 inhibitor	*Brodalumab*	2017	Psoriasis	AMAGINE-1; week 12: 83.3% [63]	AMG 827; week 16: 44% [64]	Injection-site reactions, upper respiratory infections, and nausea. Increased risk of new-onset or worsening inflammatory bowel disease (IBD) and suicide risk [48,49,50,51].
	*Bimekizumab*	2023	Both	BE OPTIMAL; week 16: 77.4% [65]	BE OPTIMAL; week 16: 44% [65]	
	*Ixekizumab*	2016	Both	UNCOVER-3; week 12: 89.1% [66]	SPIRIT-P1; week 12: 62.1% [67]	
	*Secukinumab*	2015	Both	ERASURE; week 16: 81.6% [68]	FUTURE-5; week 16: 62.6% [69]	
IL-23 inhibitor	*Guselkumab*	2017	Both	VOYAGE; week 12: 91.2% [70]	DISCOVER-2; week 24: 64% [71]	Injection-site reactions and upper respiratory infections. Increased risk of cardiovascular events, sepsis, pneumonia, cellulitis, and breast cancer [52,53,54].
	*Tildrakizumab*	2018	Psoriasis	ReSURFACE-1; week 28: 81.5% [72]	SUNPG1623; week 24: 75.5% [73]	
	*Risankizumab*	2019	Both	ULtIMMa-1; week 16: 75.3% [74]	KEEPsAKE-1; week 12: 57.3% [75]	

### 1.5. Treatment Management for Psoriasis and Psoriatic Arthritis as Comorbidities

The management of psoriasis should be personalized based on disease severity and coexisting conditions. Mild psoriasis cases are often well controlled with topical therapies, whereas more severe presentations may require phototherapy or systemic medications. Systemic therapies are also beneficial for PsA, though their effectiveness in addressing joint symptoms varies. Treatment strategies can either focus on psoriasis alone or incorporate PsA management. For individuals with mild psoriasis and mild PsA, topical corticosteroids combined with NSAIDs may be sufficient.

Screening for PsA is recommended for all psoriasis patients, particularly at initial evaluation. Those with symptoms of peripheral or axial arthritis should undergo further assessment to determine the extent of involvement across PsA domains. Mild psoriasis with mild PsA may be managed with topical corticosteroids and NSAIDs, while refractory PsA warrants a rheumatology referral and systemic therapy.

Treatment decisions should take into account the extent of skin disease, previous treatments, existing medications, comorbidities, and patient preferences. Individuals with mild to moderate psoriasis (BSA < 10%) and active PsA may begin with conventional systemic agents such as MTX alongside topical treatments [53]. In contrast, those with severe psoriasis (BSA > 10%) and PsA may require biologic therapy, including TNFi, IL-17i, IL-23i, or cytotoxic T-lymphocyte-associated-antigen-4-Ig (CTLA4-Ig) agents [53]. If a patient’s PsA does not respond to DMARDs, switching to biologic therapy is recommended rather than trying a different DMARD. TNFi is typically the first-line biologic choice unless contraindications exist, with IL-17i, IL-23i, or CTLA4-Ig available as alternative options. For those with an inadequate response to TNFi, combination therapy with MTX may improve outcomes [53].

Patients already undergoing psoriasis treatment who develop active PsA should transition to biologic therapy as per treatment guidelines, and those on biologics should be reassessed for alternative options if their condition remains uncontrolled. Apremilast and NSAIDs can be used adjunctively to address persistent symptoms. Treatment strategies should remain adaptable to individual patient needs, ensuring a holistic approach to disease management [53].

### 1.6. Unmet Needs and Challenges of Current Psoriatic and Psoriatic Arthritis Treatment Options

Current psoriatic and PsA treatment options address many aspects of the conditions, but do not address patients who continue to have active disease. One challenge is the development of anti-drug antibodies, known to occur in the TNF class, which can result in resistance and reduced efficacy of biologic therapies over time, often requiring treatment adjustments or combination approaches [76,77]. The presence of patient comorbidities, such as cardiovascular disease, metabolic syndrome, and inflammatory bowel disease, can limit the use of certain medications due to contraindications or increased risk of adverse events [78]. Medication adherence to topicals remains a concern due to the frequent application that can be burdensome for patients. Alternatively, for patients with mild psoriasis or PsA, there are currently no biologic therapies approved to expand treatment options. Emerging therapies to address this gap could offer benefits for patients with less severe disease, particularly as an alternative to topicals or for those who struggle with adherence to topical treatments. Financial barriers associated with medication costs can limit healthcare accessibility and disease management. Long-term safety outcomes of infection risk, malignancy, and organ toxicity remain an important consideration in treatment selection and monitoring.

While there are many FDA-approved medications for psoriasis, there is a greater unmet need for PsA medications. The availability of PsA-specific therapies is comparatively limited, and not all medications approved for psoriasis demonstrate equal efficacy in addressing PsA symptoms. Biologics and OSMs that are initially approved for psoriasis often subsequently receive FDA approval for PsA (and vice versa), but this is not always the case. Achieving comprehensive disease control for psoriatic disease often requires therapies that can effectively manage both skin and joint symptoms simultaneously. The need for PsA clinical trials and the development of expanded treatment options is essential to address the challenges of managing skin- and joint-related symptoms and the overall disease burden patients may encounter.

## 2. Methods

PubMed was used to search for articles published between 2019 and 2024 using the following keywords: [psoriasis treatments] AND [biologics] OR [biosimilars] OR [topicals] OR [orals]. Studies were selected based on the quality of evidence, rigor of research methods, and relevance to the topic. Priority was given to high-quality literature that provides robust and clinically significant insights into psoriasis therapies. Clinical trial data were obtained from the original PubMed search and supplemented with a search on ClinicalTrials.gov, using [psoriasis] as the condition and the generic drug name as the intervention. Only completed trials with publicly available results were included in the analysis.

## 3. Results

### 3.1. New Therapeutic Advancements for Psoriasis and PsA

Janus kinase (JAK) and tyrosine kinase (TYK) inhibitors continue to expand the therapeutic options for psoriasis and PsA (Table 4). Among JAK inhibitors, tofacitinib (a JAK1/JAK3 inhibitor) was FDA-approved for PsA in 2017 for patients with active PsA who have had an inadequate response to or cannot tolerate methotrexate or TNF inhibitors [23]. Two randomized phase 2 and 3 clinical trials had improvement in joint and skin symptoms, with 66.7% improvement by week 12 and 79.4% by week 52 [79]. Other JAK inhibitors, such as peficitinib (JAK3, JAK1, and JAK2 inhibitor), solcitinib (JAK1 and JAK2 inhibitor), and abrocitinib (JAK1 inhibitor), had moderate efficacy in early trials, with improvements ranging from 54% to 60% [80]. Baricitinib (JAK1/JAK2 inhibitor) and itacitinib adipate (JAK1 inhibitor) had limited efficacy in early studies, with minimal progression in clinical trials [81].

Emerging therapies targeting both TYK2 and JAK1 pathways, such as brepocitinib, had an increased efficacy of 86.2% improvement by week 12 in PASI [81]. Ropsacitinib (TYK2/JAK2 inhibitor) had 73.2% improvement by week 16 [81]. Among TYK2-specific inhibitors, deucravacitinib was FDA-approved in 2022 for psoriasis and achieved 75% improvement by week 12 [81]. Additional TYK2 inhibitors, such as TAK-279 (zasocitinib), had efficacy rates of 68% by week 12, with ongoing phase 3 trials [81]. Contraindications include patients with active or latent tuberculosis, history of cardiovascular diseases or thrombosis, and malignancy [87].

Retinoic acid-related orphan receptor (RORγT) inhibitors, such as VTP-43472 and JTE-451, are being investigated for their ability to regulate Th17 cell differentiation in psoriasis pathogenesis [88]. Current clinical trials face challenges with hepatic toxicity and limited efficacy [89]. Contraindications consist of patients with a history of inflammatory bowel disease, as RORγT inhibitors can cause or exacerbate the disease [90].

An oral IL-23 inhibitor, JNJ-2113, is a peptide IL-23 receptor antagonist [91]. A randomized phase 2b clinical trial had 78.6% of patients using 100 mg twice daily achieve PASI-75 at week-16 [91]. The medication was well tolerated, with mild adverse effects, including nasopharyngitis. Safety concerns include an increased risk of serious infections, cardiovascular events, and malignancy, particularly breast cancer, necessitating caution in patients with pre-existing cardiovascular disease or a history of malignancy [91]. 

DC-806 is an OSM that inhibits IL-17A [92]. A randomized phase 1 clinical trial had 43.7% of patients using 800 mg twice daily achieve PASI-75 at week 4 compared to placebo, with mild adverse events [92]. This medication is currently undergoing phase 2b for efficacy and safety. Current adverse effects include an increased risk of mucocutaneous candida infections, inflammatory bowel disease exacerbation, and major cardiovascular events, making them unsuitable for individuals with a history of inflammatory bowel disease or significant cardiovascular risk factors [92].

Sonelokimab is a trivalent nanobody that is specific to IL-17A, IL-17F, and human serum albumin [86]. In a randomized phase 2b clinical trial, 77–88% of patients using sonelokimab 120 mg had a score of 0 or 1 on the Investigative Global Assessment (IGA) scale at week 12 compared to placebo [86]. Adverse effects included nasopharyngitis, candida, Crohn’s disease, and pruritus [86]. Two randomized phase 3 sonelokimab clinical trials are currently underway for PsA for a total of 52 weeks [93]. Potential contraindications may include severe infections and hypersensitivity reactions [93].

Sphingosine-1-phosphate (S1P1R) antagonists, such as ponesimod, modulate B and T immune cell migration and are expressed in skin, lymphoid, and cardiac tissue [94]. The medication had up to 77.4% improvement by week 28, and adverse effects of hepatotoxicity, dyspnea, and dizziness were observed [95]. Cardiac monitoring is recommended due to S1P1R receptors in cardiac myocytes and risk of bradycardia [95]. Contraindications include cardiovascular conditions such as heart failure, uncontrolled hypertension, or arrhythmias, as they can impact heart rate and vascular integrity [95]. 

A3 adenosine receptor agonists, such as piclidenoson, are a class of G-protein coupled adenosine receptors, with other adenosine receptor agonists for A1, A2, and A3 [96]. A1R and A2R are associated with cardiovascular effects, while A3R had a better therapeutic and safety index and achieved modest efficacy, with 33% improvement by week 32 with no major adverse events reported [81]. A3AR agonists may be contraindicated for individuals with cardiac conduction abnormalities or significant cardiovascular disease due to their role in regulating inflammatory pathways and vascular function [81].

Heat shock protein (HSP) 90 inhibitors, such as RGRN-305, achieved PASI50 responses of 71–94% by week 12 [81]. HSP90 inhibitors have been associated with hepatic toxicity and immune suppression, making them contraindicated in individuals with liver disease or active infections [81].

Rho-associated protein kinases (ROCK-2) inhibitors, such as belumosudil, represent a novel class targeting cytokine regulation and immune signaling pathways, with early evidence suggesting their potential to improve inflammatory conditions for psoriasis and PsA [81]. Randomized phase 2 clinical trials achieved 50% in PASI by week 12 [81]. ROCK-2 inhibitors can impact vascular function and immune modulation and are contraindicated in patients with significant cardiovascular disease, hypertension, or thrombotic disorders [81]. These emerging therapies are still undergoing safety and efficacy trials, and while they offer promising new treatment options for psoriasis and PsA, their long-term risk profiles remain under investigation. These therapies reflect the growing complexity and innovation of treatments for these inflammatory conditions.

Ongoing clinical research aims to address gaps by investigating novel therapeutic agents that address unmet needs, including achieving sustained remission and improving long-term safety outcomes. Some of these emerging therapies, such as S1P receptor antagonists and A3R agonists, may require cardiac monitoring, which could limit their perception as safe options for widespread use. TYK2 inhibitors, another promising class of oral therapies, may be twice as effective as oral apremilast. However, their potential for a viral reactivation signal for herpes zoster and their impact on interferon activity raise safety concerns that may limit their use. This presents challenges in positioning these newer treatments as first-line options, particularly when compared to therapies with more favorable safety profiles.

The success of oral apremilast demonstrates the value of having oral treatments that are perceived as safe, even when tolerability issues exist. An unmet need for more effective oral therapies, such as IL-23 inhibitors and possibly IL-17 inhibitors, if they do not exacerbate or trigger inflammatory bowel disease, are well suited to fill this niche by offering higher efficacy while maintaining a manageable safety profile. Demonstrating that new treatment products are as safe or safer than IL-23 blockade will be challenging.

**Figure 1:**
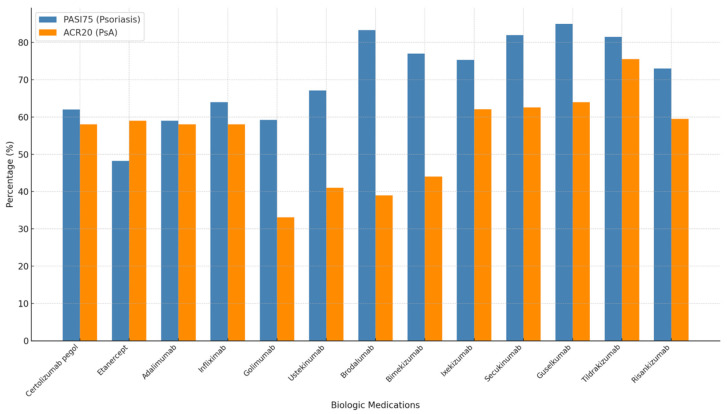
This bar graph compares the percentages of Psoriasis Area and Severity Index 75% (PASI75) and American College of Rheumatology 20% (ACR20) for biologic medications used in the treatment of psoriasis and PsA. Each medication is represented with two bars: the blue bar indicates PASI75 percentages and the orange bar indicates ACR20 percentages. Most biologic medications had higher PASI75 percentages compared to ACR20.

Biologic therapies achieve high PASI75 rates at 12–16 weeks in clinical trials, often exceeding 50%, but there remains a need for advancements in PsA treatments to enhance efficacy (Figure 1). The disparity between PASI75 and ACR20 presents an opportunity for new medications to close this gap and achieve better outcomes in managing joint symptoms associated with PsA.

### 3.2. Treatment Strategies and Considerations for Emerging New Therapies

For mild psoriasis disease, topical treatments and oral apremilast remain the primary approaches, while moderate to severe disease may require systemic treatments or phototherapy. Historically, there is poor adherence to topicals and thus there may be a role for systemic therapies in patients with objectively mild but subjectively more severe psoriasis resistant to topical treatment. Systemic therapies may also be effective in the management of PsA. Patients with psoriasis should be screened for comorbid PsA. Mild PsA symptoms, such as joint pain, can often be managed with NSAIDs without the need to escalate to systemic medications that target both conditions. In some cases, treatment may focus on managing cutaneous psoriasis independently of the arthritis, prioritizing dermatologic outcomes while addressing joint symptoms as needed. If joint inflammation becomes refractory or progresses to more severe arthritis, initiating systemic therapy and referring the patient to rheumatology are advised. Collaborating within a multidisciplinary team is essential to effectively manage the intricate dermatological and rheumatologic aspects of care for these patients. Providers should carefully consider the patient’s psoriatic skin condition, current medications, previous treatments, comorbidities, and preferences before initiating or modifying treatment plans. This individualized approach ensures that therapy aligns with the patient’s unique clinical profile and goals, optimizing outcomes and quality of life.

## 4. Conclusions

The management of psoriasis and PsA continues to evolve, with advancements and insights reshaping therapeutic approaches. The integration of OSMs, conventional DMARDs, biologics, and emerging therapies like JAK inhibitors has expanded the array of treatment options, enabling a more personalized approach for individual patient needs. Current research is exploring the efficacy and safety of combining these systemic therapies, further broadening the spectrum of available treatments. New medications targeting distinct mechanisms of the underlying inflammatory processes are in clinical trials. As these therapies demonstrate their potential, they are likely to be incorporated into future clinical guidelines, providing new pathways for effective disease management.

## Figures and Tables

**Table 1:** Topical therapeutics for psoriasis and psoriatic arthritis treatment. The discussed topical drugs are organized within categories alongside their FDA approval year.

Topical Therapies			Year FDA Approved
Steroid Topicals	Corticosteroids		
Non-steroidal topicals	Vitamin D analogs	*Calcipotriene*	1977 [13]
		*Tazarotene*	2010 [14]
		*Calcitriol*	1997 [14]
	PDE4 inhibitor	*Roflumilast*	2009 [14]
	Aryl hydrocarbon receptor antagonist	*Tapinarof*	2022 [15]
	Calcineurin inhibitors	*Tacrolimus* *Pimecrolimus*	2022 [15]
	Others	Coal tar	

**Table 2:** Oral small molecules used in the treatment of psoriasis and psoriatic arthritis. The OSM drugs discussed are categorized below and placed alongside their FDA approval year.

Oral Small Molecules (OSMs)		Year FDA-Approved	Approved for Psoriasis, PsA, or Both	Psoriasis Clinical Trial and PASI 75 Improvement	Psoriatic Arthritis Clinical Trial and ACR 20 Improvement	Adverse Events
Immunosuppressant	*Methotrexate*	1972	Psoriasis	Tight control of psoriatic arthritis (TICOPA); week 12: 27.2% [29]	Tight control of psoriatic arthritis (TICOPA); week 12: 40.8% [29]	Hepatotoxicity, GI upset, myelosuppression [29]
	*Cyclosporine A*	1997	Psoriasis	16 randomized clinical trials; week 12: 50–97% [30]	Randomized clinical trial; week 48: 47% [31]	Nephrotoxicity, hypertension, hyperuricemia, hyperglycemia, neurotoxicity [30]
Vitamin A derivative	*Acitretin*	1996	Psoriasis	Randomized clinical trial; week 8: 23% [32]	NA	Hepatotoxicity, teratogenicity, hyperlipidemia [32]
PDE4 inhibitor	*Apremilast*	2014	Both	ESTEEM 1–2; week 16: 33% [33]	PALACE 1,2,3; week 16: 38% [34]	GI upset, headache, weight loss [34]
JAK/TYK2 inhibitors	*Tofacitinib*	2017	PsA	Randomized clinical trial; week 12: 66.7% [35]	Randomized clinical trial; week 12: 65.7% [36]	Headache, diarrhea [35]
	*Upadacitinib*	2021	PsA	Randomized clinical trial; week 12: 52.3% [37]	Randomized clinical trial; week 12: 71% [38]	Upper respiratory infection, myelosuppression, rash [38]
	*Deucravacitinib*	2022	Psoriasis	POETYK PSO-1 week 16: 58.4% [39]	Randomized clinical trial; week 12: 62.7% [40]	Myalgia, fatigue, fever [39]

**Table 4:** Current ongoing clinical trial therapies for psoriasis and PsA.

Therapy Type	Drug Name	Mechanism of Action	Efficacy (PASI Improvement)	Adverse Effects
JAK Inhibitors	Tofacitinib	JAK1 and JAK3 inhibitor	Week 12: 66.7%, Week 52: 79.4% [82]	Cytopenia, infections [82]
	Peficitinib	JAK3, JAK1, and JAK2 inhibitor	Week 6: 58.8% [83]	Nasopharyngitis, diarrhea [83]
	Solcitinib	JAK1 and JAK2 inhibitor	Week 12: 57% [84]	Headache, fatigue [84]
	Abrocitinib	JAK1 inhibitor	Week 4: 60% [81]	Nausea, neutropenia [81]
TYK2 Inhibitors	Deucravacitinib	TYK2 inhibitor	Week 12: 75% [85]	Nasopharyngitis [85]
	TAK-279 (zasocitinib)	TYK2 inhibitor	Week 12: 68% [74]	Cytopenias, acne [74]
	ESK-001	TYK2 inhibitor	Week12: 58.7% [81]	Nasopharyngtitis, acne, headache, urinary tract infection [81]
RORγT Inhibitors	VTP-43472	Regulates Th17 differentiation	Week 4: 30% [81]	Hepatic toxicity [81]
	JTE-451	Regulates Th17 differentiation	Week 16: 22% [81]	Not reported
Oral IL-23 inhibitor	JNJ-2113	IL-23 inhibitor	Week 16: 78.6% [81]	Nasopharyngitis [81]
Oral IL-17 inhibitor	DC-806	IL-17A inhibitor	Week-4: 43.7% [81]	No major adverse events [81]
Nanobody	Sonelokimab	IL-17A, IL-17F, and human serum albumin	Week-12: 77–88% (IGA score of 0 or 1) [86]	Nasopharyngitis, candida, Crohn’s disease, and pruritus [86]
S1P1R Antagonists	Ponesimod	Modulator of S1P1R	Week 16: 48.1%, Week 28: 77.4% [81]	Dyspnea, liver enzyme abnormalities, cardiac monitoring [81]
A3AR Agonists	Piclidenoson	Agonist of A3AR	Week 32: 33% [81]	Nasopharyngitis, Cardiac monitoring [81]
HSP90 Inhibitors	RGRN-305	Inhibition of HSP90	Week 12: 71–94% (PASI50) [81]	Exanthematic reactions [81]
ROCK-2 Inhibitors	Belumosudil	Inhibition of ROCK-2	Week 12: 16.7% (PASI50) [81]	Diarrhea [81]

## Data Availability

Data sharing is not applicable to this article as no datasets were generated or analyzed during the current study.
